# Profiling bacterial communities associated with sediment-based aquaculture bioremediation systems under contrasting redox regimes

**DOI:** 10.1038/srep38850

**Published:** 2016-12-12

**Authors:** Georgina Robinson, Gary S. Caldwell, Matthew J. Wade, Andrew Free, Clifford L. W. Jones, Selina M. Stead

**Affiliations:** 1 School of Marine Science and Technology, Newcastle University, Newcastle, NE1 7RU, UK; 2 Department of Ichthyology and Fisheries Science, Rhodes University, Grahamstown 6140, South Africa; 3 School of Civil Engineering and Geosciences, Newcastle University, Newcastle, NE1 7RU, UK; 4 Institute of Quantitative Biology, Biochemistry and Biotechnology, School of Biological Sciences, University of Edinburgh, Edinburgh, EH9 3FF, UK

## Abstract

Deposit-feeding invertebrates are proposed bioremediators in microbial-driven sediment-based aquaculture effluent treatment systems. We elucidate the role of the sediment reduction-oxidation (redox) regime in structuring benthic bacterial communities, having direct implications for bioremediation potential and deposit-feeder nutrition. The sea cucumber *Holothuria scabra* was cultured on sediments under contrasting redox regimes; fully oxygenated (oxic) and redox stratified (oxic-anoxic). Taxonomically, metabolically and functionally distinct bacterial communities developed between the redox treatments with the oxic treatment supporting the greater diversity; redox regime and dissolved oxygen levels were the main environmental drivers. Oxic sediments were colonised by nitrifying bacteria with the potential to remediate nitrogenous wastes. Percolation of oxygenated water prevented the proliferation of anaerobic sulphate-reducing bacteria, which were prevalent in the oxic-anoxic sediments. At the predictive functional level, bacteria within the oxic treatment were enriched with genes associated with xenobiotics metabolism. Oxic sediments showed the greater bioremediation potential; however, the oxic-anoxic sediments supported a greater sea cucumber biomass. Overall, the results indicate that bacterial communities present in fully oxic sediments may enhance the metabolic capacity and bioremediation potential of deposit-feeder microbial systems. This study highlights the benefits of incorporating deposit-feeding invertebrates into effluent treatment systems, particularly when the sediment is oxygenated.

Bacterial communities present in marine sediments play significant ecological and biogeochemical roles in organic matter decomposition and nutrient cycling. Abiotic and biotic factors including the chemical environment of sediments (e.g. reduction-oxidation potential) and grazing can significantly influence bacterial communities^1^,^2^. Deposit-feeding macrofauna further affect sediment microbiology through burying activities (bioturbation) that reworks the sediment and enhances sediment-water exchange, stimulating bacterial production and net mineralization rates^3^. Tight coupling therefore exists between bioturbation, redox conditions, microbial communities, and detritus processing^4^. To date, a lack of understanding of the complex interactions between bacteria and deposit-feeders has created a knowledge gap in microbial mineralization within aquaculture systems. Information on this relationship offers huge potential to optimise future designs for sustainable aquaculture production technologies.

In intensive aquaculture systems, high organic loading rates and/or the accumulation of solid wastes can frequently exceed the microbial mineralization capacity of the system^5^. This can produce water column hypoxia and the release of toxic metabolites (e.g. ammonia, nitrite, hydrogen sulphide), which negatively impact the health and survival of the farmed species. Deposit-feeding sea cucumbers are the focus of growing attention as potential bioremediators in aquaculture systems due to their ability to convert faeces and waste feed into high value secondary biomass^6^,^7^,^8^.

*In situ* bioremediation technologies frequently employ the addition of external electron acceptors, most frequently oxygen, to enhance the aerobic decomposition of organic matter and alleviate the constraints imposed by the naturally slow mineralization process in sediments^9^. The percolation of oxygenated water, one of the most cost-effective approaches currently used for *in situ* sediment remediation, is applicable to the development of aquaculture bioremediation systems that integrate epibenthic deposit-feeders.

Combining bioremediation technologies (increasing oxidant supply) with the production of high value secondary livestock (e.g. sea cucumbers) grown on aquaculture effluents remains a largely novel and unexplored concept. Robinson *et al*.^10^ investigated the effects of manipulated sediment culture systems, describing either fully oxic or redox-stratified (oxic-anoxic) sediments, on the growth and biomass carrying capacity of the sea cucumber *Holothuria scabra*. Sea cucumbers reared on fully oxic sediments experienced stunted growth and yielded a lower biomass relative to those reared on oxic-anoxic sediments (which mirror the natural habitat of *H. scabra*). The active circulation of oxygenated water successfully maintained sediment under fully oxic conditions and appeared to increase the rate of organic matter degradation. We hypothesised that the carbon oxidation and nitrogen cycling conditions within the contrasting redox regimes affected both the quality and quantity of food resources available for deposit-feeder growth. We further hypothesised that the oxic-anoxic sediments would harbour microbial communities that were dominated by heterotrophic bacteria operating anaerobic and fermentative metabolisms. In theory, this should provide a steady release of more nutritionally favourable food resources for deposit-feeders than fully aerobic systems.

A number of studies have used next-generation sequencing to investigate bacterial community composition in the sediments of sea cucumber aquaculture ponds and adjacent natural habitats^11^,^12^; however, to date, no study has investigated the effect of oxygen supply on sediment microbial composition and community structure in aquaculture systems with a view to improving their bioremediation capacities. An exploration of the mechanisms by which bacterial community composition is affected by abiotic and biotic factors could contribute to improving our understanding of aquaculture effluent treatment systems. Currently, the relationships between bacterial community structure and the redox regime of marine sediments are poorly understood^13^. Therefore, it is important to investigate the effect of oxygen availability on the structure and functional potential of bacterial communities in sediment-based bioremediation systems^14^. This is a timely study due to the high level of interest from governments worldwide to increase aquaculture production to address global food security issues, including the provision of cost-effective and sustainable aquaculture waste treatment solutions.

This study presents novel findings that advance observations detailed in Robinson *et al*.^10^ by characterising the diversity, structure and predicted metabolic functions of the microbial communities present in the sediment of *H. scabra* culture tanks subjected to contrasting redox regimes (oxic and oxic-anoxic). The environmental drivers behind changes in the microbial communities are evaluated and discussed in relation to the use of deposit-feeders for bioremediation purposes.

## Results

### Water and sediment quality

The water temperature in the oxic-anoxic treatment (29.81 ± 0.01 °C) was significantly higher than in the oxic treatment (29.13 ± 0.12 °C; Student’s t-test, t = 5.84, p = 0.004), whereas dissolved oxygen concentrations were significantly higher in the oxic treatment tanks (7.89 ± 0.06 mg L^−1^ versus 7.49 ± 0.11 mg L^−1^; Student’s t-test, t = −3.15, p = 0.035). The redox potential at the base of the sediment was significantly different between treatments (Student’s t-test; t = −13.93, p = 0.0002; Table 1); the oxic-anoxic sediment had a negative redox potential (−188.42 ± 11.52 mV), indicating predominantly reduced conditions, contrasting with the sediment in the oxic treatment which was 33.50 ± 11.00 mV, indicating predominantly oxic conditions. Also, the oxic treatment produced more than double the cyanobacterial biomass (221.61 ± 34.95 g compared with 99.66 ± 2.72 g dry weight) than the oxic-anoxic treatment (Student’s t-test, t = −3.48, p = 0.025).

### *Holothuria scabra* survival and growth

**Figure 1:**
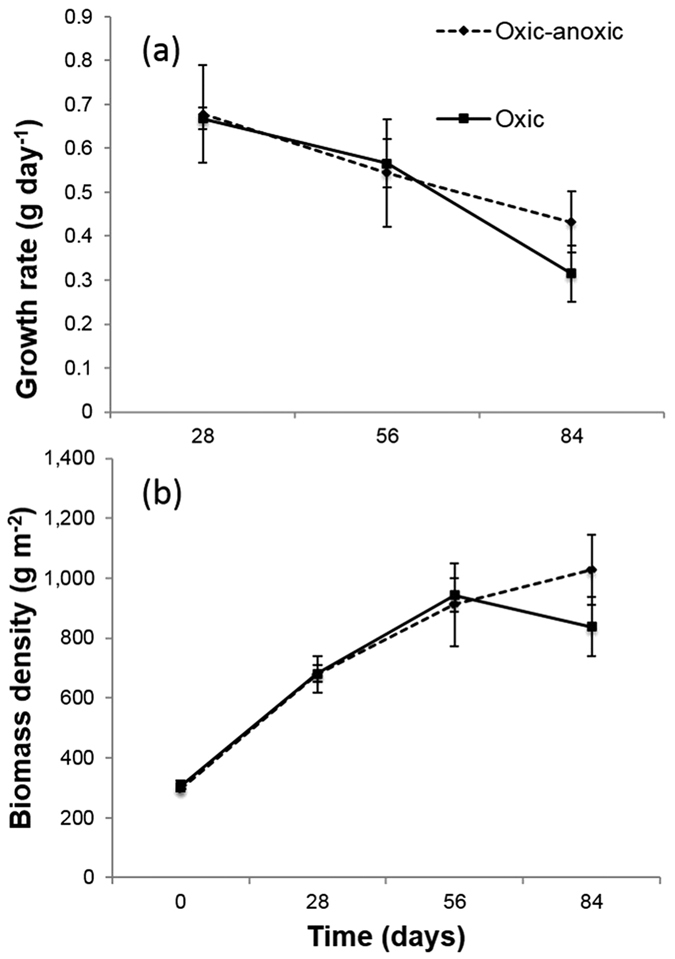
(**a**) The mean (±standard error) growth rate and (**b**) the mean (±standard error) biomass density of *Holothuria scabra* (n = 4) reared in tanks with either a stratified oxic-anoxic and fully oxic sand sediment.

Survival was 100% in all treatments. The mean wet weight (±standard error) was similar in both treatments at the start of the trial (7.57 ± 0.27 g individual^−1^, Student’s t-test; t = −2.03, p = 0.11). Growth rates in both treatments were positive throughout the duration of the trial, however the rate decreased over time. The biomass density increased linearly in both treatments up to Day 56; however, it began to decrease in the oxic treatment during the final third of the trial. There was no significant difference in mean growth rate after 84 days (t-test; t = 1.24, p = 0.28; Fig. 1a). Sea cucumbers in the oxic-anoxic treatment achieved a final mean density of 1028.50 ± 117.46 g m^−2^ compared with 837.96 ± 99.70 g m^−2^ in the oxic treatment (Fig. 1b).

### Sequencing and quality control

Pyrosequencing of 16 S rRNA gene V4–5 amplicons yielded 72,675 reads; however, due to a low abundance of reads, three samples (replicate A from oxic-anoxic 0 cm, and replicates A and B from oxic 4 cm) were removed from further analysis. Subsequent to quality control, primer trimming, size exclusion, and removal of unassigned bacteria and archaea, a total of 47,573 optimised reads from the 15 samples remained. Sequences were subsampled to 1,264 (the minimum number of sequences in all samples).

### Comparison of bacterial community composition between treatments

Rarefaction curves indicated that the oxic treatment was not sampled to saturation whereas sequencing depth was sufficient for the oxic-anoxic treatment (Supplementary Fig. 1). The richness estimators and diversity indices were all significantly higher in the oxic treatment (Table 2) indicating that the sediments maintained under a fully oxic redox regime harboured more diverse and stable bacterial communities than the stratified oxic-anoxic sediments.

**Figure 2:**
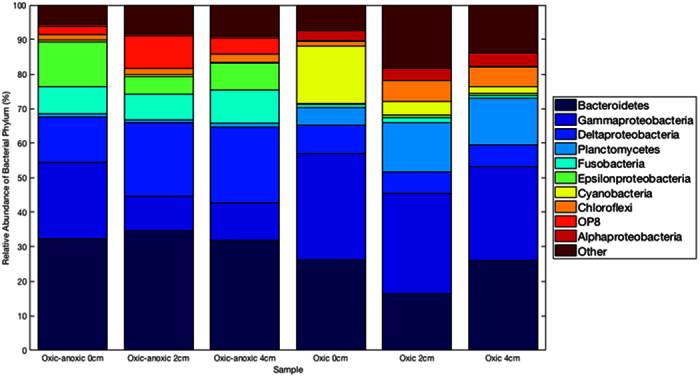
The relative abundance of the bacterial reads classified at phylum level (including Proteobacteria sub-classes) from the different sediment redox regimes and depths. Each bar represents the mean of treatment replicates (n = 3).

There were 20 unique phyla, 21 candidate divisions, and two phyla proposed by the Greengenes database ([Caldithrix] and [Thermi]). Bacteroidetes had the highest sequence abundance representing 27.83 ± 2.04% (n = 15) of all sequences, followed by Gammaproteobacteria (20.92 ± 2.52%), Deltaproteobacteria (13.74 ± 2.16%), Planctomycetes (5.25 ± 1.52%), Fusobacteria (5.02 ± 1.56%), Epsilonproteobacteria (4.53 ± 1.42%) and Cyanobacteria (4.52 ± 1.79%; Fig. 2). Unclassified bacteria (2.61% of sequences) were removed to facilitate downstream analyses. The phylum Nitrospira, the candidate divisions AncK6, GAL15, SBR1093, TM7 and the Proteobacteria sub-class TA18 were only present in oxic sediments. Similarly, phylogenetic groups from the phyla Thermotogae, Fibrobacteres, [Thermi] and the candidate divisions KSB3 and LCP-89 were only present in the oxic-anoxic sediments.

**Figure 3:**
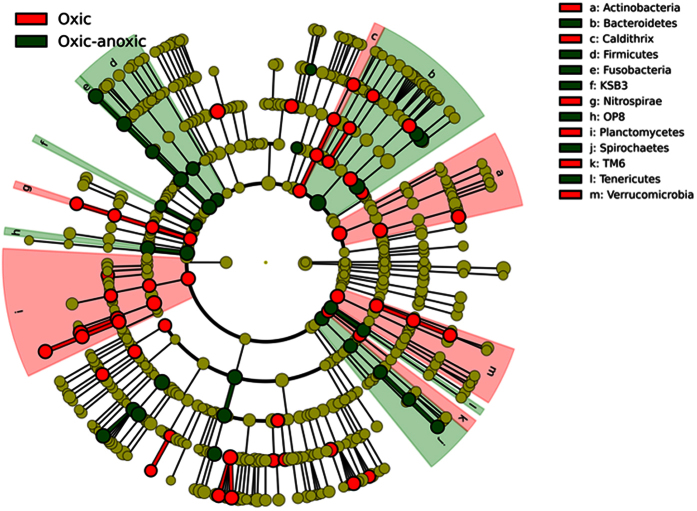
The phylogenetic distribution of microbial lineages associated with the two different sediment redox regimes (oxic-anoxic and oxic). Lineages with linear discriminant analysis (LDA) values of 5.0 or higher as determined by effect size measurements (LEfSe) are displayed. The six rings of the cladogram stand for domain (innermost), phylum, class, order, family and genus. Enlarged circles in dark green and red are differentially abundant taxa identified as taxonomic biomarkers in the two different redox regime treatments (red = oxic-anoxic sediment, green = oxic sediment). Light green circles are biomarkers with LDA scores of less than 5.0. Labels are shown at the phylum level only.

**Figure 4:**
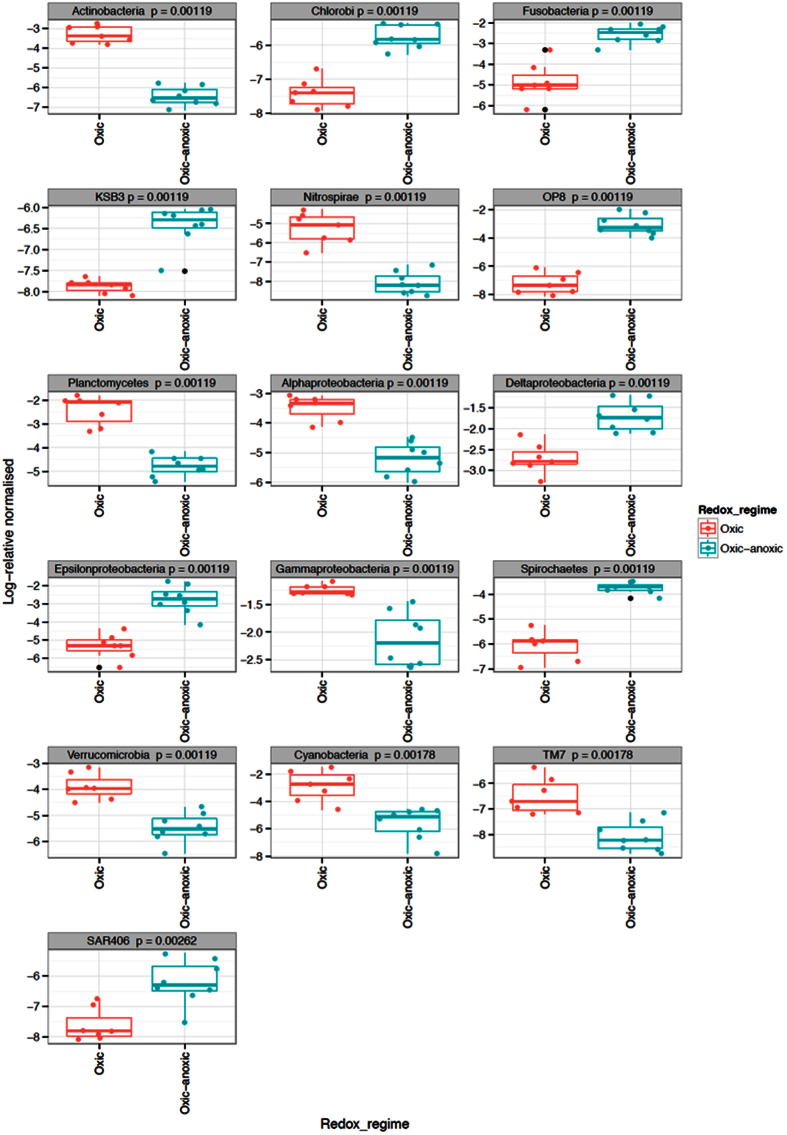
Bacterial phyla with significantly different relative abundances between the oxic-anoxic and oxic redox regimes (Kruskal-Wallis test). Data are presented as log normalised relative abundances.

**Figure 5:**
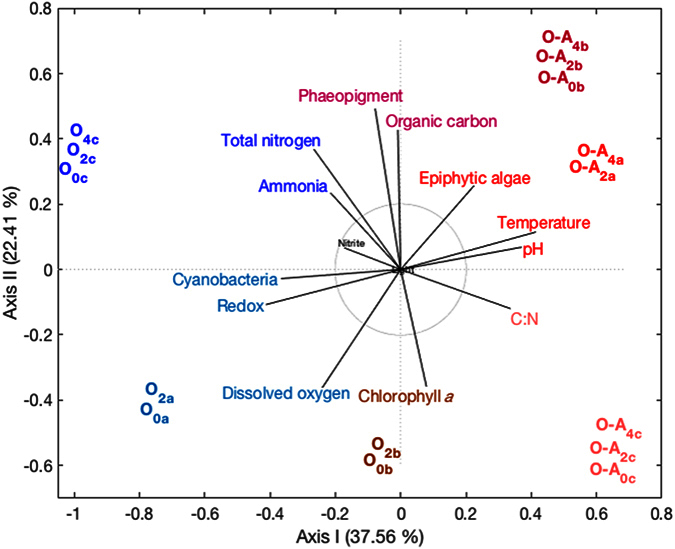
A principal components analysis biplot of the correlation between the bacterial community composition and the environmental parameters plotted as vectors.

The sediment redox regime led to significant differences in the relative abundance of eight phyla, five candidate divisions and four of the Proteobacteria classes. Figure 3 presents a taxonomic representation of the statistically significant relevant biomarkers that were identified in the oxic-anoxic (red) and oxic (green) treatments. There was a significantly higher number of sequences classified within the phyla Actinobacteria, Cyanobacteria, Nitrospirae, Planctomycetes, Verrucomicrobia, the Alpha-, and Gammaproteobacteria sub-classes and the candidate division TM7 in the fully oxic sediments, while the number of sequences within the Chlorobi, Fusobacteria, Spirochaetes, the Delta-, and Epsilonproteobacteria sub-classes and the candidate divisions H-178, KSB3, OP8 and SAR406 were significantly higher in the oxic-anoxic sediments (p < 0.01; Fig. 4). Beta diversity analysis performed at the phylum level revealed that the bacterial communities were distinct between the oxic and oxic-anoxic treatments. The samples clustered into two clear groups by treatment along axis 1 that explained 37.56% of the variation, while samples were structured by sediment depth along axis 2 (22.41%; Fig. 5). Sediment redox potential was the only significant environmental variable driving differences in bacterial community structure between treatments (Supplementary Table 1).

### Microbial biomarker discovery

Eighty six taxonomic biomarkers from phylum to genus level identified by Linear Discriminant Analysis Effect Size (LEfSe)^15^ with linear discriminate analysis (LDA) scores greater than 5.0 were distinguishable between the treatments. The only taxa containing biomarkers specific to both treatments were the phyla Chloroflexi, Plantomycetes, and the class Deltaproteobacteria. Fifty were identified as consistently statistically different in the oxic treatment compared with 36 in the oxic-anoxic treatment. The biomarkers enriched in the oxic treatment were classified within the phyla Actinobacteria, Bacteroidetes, Caldithrix, Chloroflexi, Cyanobacteria, Nitrospirae, Planctomycetes, Verrucomicrobia; the classes Alpha- Delta- and Gammaproteobacteria and the candidate division TM6. The oxic-anoxic treatment had 16 biomarkers classified within eight phyla, including; Bacteroidetes, Chlorobi, Chloroflexi, Firmicutes, Fusobacteria, Planctomycetes, Spirochaetes, Tenericutes, the subclasses Delta-, and Epsilonproteobacteria and the three candidate divisions H-178, KSB3, and OP8 (Fig. 4).

Classification of the bacterial biomarkers according to their oxygen-related physiology highlighted clear differences between treatments (Fig. 4). The majority of biomarkers in the oxic-anoxic treatment were strict or obligate anaerobes, whereas all of the biomarkers in the fully oxic sediments were obligate or facultative aerobes. Similarly, classification based on the type of dissimilatory metabolism revealed clear differences in the metabolic capacity and putative functional roles between treatments. Biomarkers significantly enriched in the oxic treatment covered a broad spectrum of dissimilatory metabolisms including heterotrophs, taxa with aerobic and anaerobic respiratory and fermentative metabolisms; chemolithotrophs; methylotrophs and phototrophs performing both oxygenic and anoxygenic photosynthesis (Supplementary Table 2). In contrast, all biomarkers enriched in the oxic-anoxic treatment were heterotrophs (Supplementary Table 3).

### Predicted metagenome

The PICRUSt metagenome predictions had NSTI scores ranging from 0.11 to 0.21 with an overall mean of 0.16 ± 0.01; which is comparable to the accuracy for soil samples^16^. Redox regime significantly affected the prediction accuracy, with lower NSTI scores and higher sequence similarity (hence better accuracy) to bacterial genomes for the oxic-anoxic treatment (Mixed model ANOVA; F_(1,12)_ = 60.33, p > 0.001; Supplementary Table 4). The lower degree of accuracy for the oxic sediments may be due to higher bacterial diversity and increased abundance of bacteria that do not yet have sequenced representatives.

**Figure 6:**
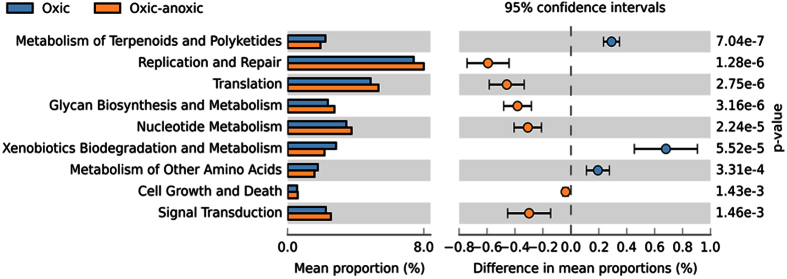
The mean proportion (%) and the difference in the mean proportion of gene counts at level two of the BRITE functional hierarchy between oxic-anoxic and oxic treatments with 95% confidence intervals. Significant differences in gene abundances were determined using two-sided Welch’s t-tests (alpha = 0.05) with a Bonferroni multiple test correction to control for false discovery rate.

A total of 236 pathways were indicated at the three-tier level of functional categories defined by the BRITE hierarchy. There were significant differences between treatments in the predicted bacterial metagenome at the two-tier level. At the top level of pathways modules, genes involved with metabolism were significantly enriched in the oxic sediment. At the functional subcategory level, three pathways within metabolism (i. xenobiotics biodegradation and metabolism; ii. metabolism of terpenoids and polyketides, and; iii. metabolism of other amino acids) were enriched in the oxic treatment. At the functional subcategory (level two), the oxic-anoxic treatment was significantly enriched with genes involved in: cell growth and death, signal transduction, translation, replication and repair, glycan biosynthesis and metabolism, and nucleotide metabolism (Fig. 6).

The higher resolution analysis of the predicted functional capacities (Supplementary Fig. 2) followed a similar trend with a predominance of orthologous genes involved in metabolism and degradation pathways enriched in the oxic sediments. Of the 28 predicted functional pathways that had significantly higher gene counts in the oxic sediments five included genes involved in xenobiotics biodegradation including fluorobenzoate, chlorocyclohexane and chlorobenzene, polycyclic aromatic hydrocarbons, naphthalene, aminobenzoate and the degradation of terpenoids and polyketides. In contrast, the bacterial communities in the redox-stratified sediments had significantly higher abundance of genes predicted to be involved in purine and pyrimidine (nucleic acid metabolism), nitrogen metabolism, carbon fixation pathways and a variety of functional pathways categorised within genetic information processing and cellular processes.

## Discussion

Analysis of 16 S rRNA gene sequences revealed highly differentiated microbial communities between the manipulated sediment systems (oxic and oxic-anoxic treatments). Redox potential was the principal driver of shifts in bacterial community composition and predicted functional capacity. However, these distinctions did not translate into significant differences in terms of sea cucumber growth as previously observed^10^.

In the present study, the final sea cucumber biomass densities were significantly higher than those achieved in Robinson *et al*.^10^ (1028.50 ± 117.46 versus 626.89 ± 35.44 g m^−2^ in the oxic-anoxic treatment, and 837.96 ± 99.70 versus 454.84 ± 14.30 g m^−2^ in the oxic treatment). The growth curves between the studies followed the same pattern, i.e. initially equal between treatments before declining more steeply under fully oxic conditions. Due to facilities access constraints the current trial was terminated prior to attaining statistical significance, however the similarities between the growth profiles give us cause to be confident that, had the trial been run for longer, a significant difference would have been achieved. The growth rate and final biomass density differences between studies can be attributed to seasonal differences between the respective trials. The Robinson *et al*.^10^ study ran from the end of the austral summer into mid-winter, whereas the current trial ran from the start of the summer and benefitted from longer day lengths (11.45 to 14.20 daylight hours compared to 10 hours), stronger irradiances and significantly higher ambient temperatures in the culture facility (mean temperatures of 29.49 ± 0.09 versus 25.71 ± 0.05 °C). Temperature is one of the key environmental variables affecting the activity, metabolism and growth rate of marine invertebrates, including *H. scabra*^17^,^18^. The longer day lengths coupled with stronger irradiances will have increased photoautotrophic production in the current study relative to Robinson *et al*.^10^, which may potentially have made more refractory carbon available to the sea cucumbers in the current study.

The active circulation of oxygenated seawater through the sediment in the oxic treatment led to the establishment of a microbial community characterised by a higher relative abundance, diversity, richness and evenness, with an enrichment of predicted metabolic and functional potential for bioremediation. This contrasts the bacterial communities present in the predominately anaerobic sediment of the oxic-anoxic treatment (Supplementary Tables 3 and 4). The presence of molecular oxygen is a major factor determining changes in bacterial community structure with consequent impacts on biogeochemical cycles and organic matter mineralization^1^,^13^,^19^,^20^,^21^,^22^. Obligate aerobes and microaerophiles require oxygen for energy production and depend on the transfer of electrons to oxygen, which is the final electron acceptor in electron transport-linked oxidative phosphorylation. All of the bacterial biomarkers in the fully oxic treatment were obligate or facultative aerobes. In the redox-stratified sediments the molecular diffusion of oxygen would have been limited to a depth of a few millimetres below which microbial activity would have been predominately anaerobic^23^,^24^. This was supported by the majority of biomarkers being classified as either strict or obligate anaerobes.

Competition between bacteria is based on substrate uptake kinetics and the efficiency with which the substrate is coupled to growth^25^. Carbon is more efficiently incorporated during aerobic respiration resulting in faster growth rates^26^. The form of dissimilatory metabolism has a large impact on bacterial growth rate and their dominance in the community. The higher evenness in the oxic treatment suggests that the bacterial community was more stable^27^.

Within redox-stratified sediments bacteria with very different metabolic capacities are distributed according to redox potential and the spatial distribution of electron acceptors. The differing transport regimes between oxic (advective) and oxic-anoxic treatments (molecular diffusion) will have differentially affected microbial activity. The active circulation of oxygenated water in the oxic treatment will have accelerated the exchange of pore water with the overlying water column and increased the rates of nutrient supply and removal of waste products to and from the bacteria. The accumulation of metabolic waste products can inhibit metabolic pathways, thus their removal and re-distribution for assimilation by other species is an important process^25^,^28^.

Interestingly, genes for xenobiotics degradation and metabolism were predicted to be enriched in the oxic treatment. The successful bioremediation of aquaculture wastes involves the combined actions of a diverse consortium of heterotrophic, chemolithotrophic and phototrophic bacteria^5^. In the oxic treatment, a high diversity of dissimilatory metabolisms was indicated, including taxa with heterotrophic metabolism, including aerobic and anaerobic respiration and fermentation; chemolithotrophic; methylotrophic and phototrophic metabolisms, performing both oxygenic and anoxygenic photosynthesis. In contrast, biomarkers enriched in the oxic-anoxic treatment exhibited limited functional diversity, representing only chemoorganotrophic (heterotrophic) bacteria, lending support to the previous conclusion that redox-stratified sediments may exhibit limited bioremediation potential^10^.

Particulate organic wastes comprising faeces and waste feed comprise the bulk of aquaculture effluents in recirculating aquaculture systems. To minimize sludge accumulation, a broad spectrum of heterotrophic bacteria with a good enzymatic capacity and ability to multiply rapidly is essential to maximise rates of carbon mineralization to carbon dioxide^5^. In the oxic treatment the diverse heterotrophic community could utilise the full spectrum of carbon oxidation pathways including aerobic, anaerobic and fermentative respiration. The majority of biomarkers within the phyla Bacteroidetes, Planctomycetes, Verrucomicrobia, the sub-classes Delta-, and Gammaproteobacteria, were classified as taxa with aerobic metabolisms capable of oxidising a range of complex polymeric carbon compounds (including sugars, alcohols, organic acids, amino acids and carbohydrates); underscoring the enhanced capacity for organic matter degradation in oxic sediments^29^.

Bioremediation of nitrogenous compounds relies on maximising chemolithotrophic processes that remove potentially toxic compounds (ammonia and nitrite) via nitrification, which is mediated in a step-wise process by ammonia-oxidising bacteria (AOB) and nitrite oxidising bacteria (NOB). The phylum Nitrospirae, containing chemolithotrophic bacteria that oxidise nitrite to nitrate, had a significantly higher relative abundance in the oxic treatment (Fig. 3) and was enriched to family level (*Nitrospiraceae*), present at 2 and 4 cm depths, but absent from the oxic-anoxic treatment. Similarly, AOB within the family *Nitrosomonadaceae* (Betaproteobacteria) were present in the oxic treatment at 2 and 4 cm depths, and although not identified as a biomarker, it does indicate that nitrification was occurring in the fully oxic sediment. Denitrifying bacteria are also considered important, particularly in re-circulating aquaculture systems where nitrate accumulates due to the high nitrification capacity of biofilters. Due to the lack of resolution in identifying bacterial taxa to genus level, biomarkers known to be involved in denitrification were not identified; however, sequences assigned to the family *Pseudomonadaceae* (Gammaproteobacteria), which contains denitrifying and N_2_-fixing bacteria, were present in both treatments.

Additional chemolithotrophic biomarkers involved in biogeochemical cycles included an enrichment of ferrous iron oxidisers (Acidomicrobiales) and the sulphur oxidising genus *Thiopilula* (Thiotrichales) in the oxic treatment^30^. Sulphur cycling in aquaculture systems is important as un-ionised dissolved hydrogen sulphide (H_2_S) is extremely toxic to many aquatic organisms, even at natural levels^31^. It is particularly relevant for sediment-based aquaculture systems as sulphate reduction can account for over 50% of organic matter degradation in marine sediments, leading to the production of considerable quantities of H_2_S^32^. In shrimp aquaculture, phototrophic purple and green sulphur bacteria that perform anoxygenic photosynthesis at low light intensities under anaerobic conditions are frequently mass cultured and applied to ponds to bioremediate H_2_S^5^. The oxic treatment contained two anoxygenic photosynthetic biomarkers that are capable of H_2_S oxidation, represented by purple sulphur bacteria within the family *Ectothiorhodospiraceae* (Gammaproteobacteria) and purple non-sulphur bacteria within the family *Rhodobacteracae* (Alphaproteobacteria).

In addition, phototrophic cyanobacteria performing oxygenic photosynthesis had a significantly higher relative abundance in the oxic treatment. Cyanobacterial orders that predominated in the surface sediment layers in the oxic treatment included *Oscillatoriophycideae* and *Synechococcophycideae*, contributing towards *in situ* primary production and increasing the availability of natural food resources, which is another important process in aquaculture bioremediation systems^5^. Finally, aquaculture bioremediation technologies rely on the maintenance of a diverse and stable community where undesirable species are not dominant^5^. In the oxic-anoxic treatment taxonomic biomarkers included the class Mollicutes (phylum Tenericutes), which are commensals, parasites or pathogens of a wide range of vertebrate, insect, and plant hosts^33^. In contrast, bacteriolytic taxa were represented by the family *Nannocystaceae* (order Myxococcales) in the oxic treatment that may play a role in suppressing unwanted organisms^33^.

All of the biomarkers enriched in the oxic-anoxic treatment were chemoorganotrophs, a group that obtains both carbon and energy for biosynthetic reactions from organic compounds and mediate the decomposition of organic matter in anoxic sediments^34^. The majority of biomarkers in the oxic-anoxic treatment were anaerobes, with metabolisms based on anaerobic respiration, fermentation and phototrophy (anoxygenic photosynthesis). Only two biomarkers were classified as obligate aerobes or microaerophiles; the genus SJA-88, within the proposed order Leptospirales (phylum Spirochaetes), which utilizes long-chain fatty acids or long-chain fatty alcohols as carbon and energy sources^35^; and the microaerophilic *Heliobacteraceae* which obtain energy from amino acids or the tricarboxylic acid cycle intermediates and reduce fumarate to succinate^35^,^36^. Strictly anaerobic bacteria in the oxic-anoxic treatment included the class Mollicutes (phylum Tenericutes). The remainder of biomarkers were sulphate-reducers within the family *Desulfobacteracae* and order Desulfobacterales. Most members of the family *Desulfobulbaceae* are incomplete oxidizers that form acetate as an end product, which is in turn utilised by *Desulfobacter* as the preferred general electron donor and carbon source, being oxidised completely to CO_2_^37^. A representative of the green sulphur bacteria, the Chlorobi clade OPB56, which performs anoxygenic photosynthesis and metabolizes small organic molecules, was also identified as a biomarker in the oxic-anoxic treatment^38^.

Although sulphate-reducing bacteria are important in the oxidation of organic carbon in marine sediments, these bacteria have no capacity to hydrolyse particulate organic matter, therefore, other bacteria capable of performing these complex hydrolyses are needed^39^,^40^. The majority of biomarkers in the oxic-anoxic treatment possessed a fermentative-type metabolism, including the saccharolytic Bacteroidales and Spirochaetales, which ferment carbohydrates; cellulolytic Clostiridiales which ferment cellulose; the class Phycisphaerae and candidate phylum KSB3 which ferment sugars (Supplementary Table 3). The only biomarker identified to genus level with a fermentative metabolism was *Propionigenium* (Fusobacteria), which are well adapted to marine anoxic sediments since their metabolism is based on sodium ions as coupling ions in energy conservation, preferentially using dicarboxylic acids as substrates^41^. This example serves to illustrate how the concerted actions of a consortium of bacteria are necessary for the complete mineralization of organic matter under anaerobic conditions.

The predominately anaerobic mineralization conditions in the redox-stratified sediment, which mirrors *H. scabra*’s natural habitat, likely provided a steady release of bioavailable food resources^14^ that supported the significantly higher sea cucumber biomass observed in the oxic-anoxic treatment in Robinson, *et al*.^10^, a trend which was generally supported by the current study. Anaerobic respiration produces considerable amounts of extracellular, low molecular weight organic compounds^42^ as complex polymeric molecules are stepwise split into water-soluble monomers, such as amino acids, monosaccharides, organic acids, and fatty acids^43^, which serve as substrate for fermentation. In fermentation, energy is conserved by substrate level phosphorylation and the redox balance is achieved by the excretion of reduced substances such as fatty acids and organic acids, including lactic, formic, acetic, propionic and butyric acids and H_2_, produced as the end products of catabolism. Fermentation products are energy rich due to the presence of phosphate bonds or a molecule of coenzyme A, and may be important for deposit-feeder nutrition^44^. Low molecular weight organic compounds may be adsorbed to extracellular polymers or inorganic sediment particles and thus become available to deposit-feeders by direct ingestion^14^. Uptake of dissolved organics may also occur across the epithelium^26^,^45^ or via the respiratory trees since aspidochirotid sea cucumbers that possess respiratory trees are nutritionally bipolar, possessing an ability to anally suspension feed^46^.

Successful bioremediation of aquaculture wastes involves the combined actions of a diverse consortium of functional groups of bacteria^5^. Specifically, aquaculture bioremediation technologies aim to optimise: (1) carbon mineralization to minimize sludge accumulation; (2) nitrification rates to maintain a low ammonia concentration; (5) primary productivity to stimulate carbon fixation; and (6) the maintenance of a diverse and stable community where undesirable species do not become dominant^5^. Analysis of the taxonomic composition and functional diversity of bacteria communities according to their oxygen-related ecophysiology and dissimilatory metabolism demonstrated that the oxic treatment contained all of the requisite functional groups for successful bioremediation performance.

This study demonstrates support for the theory that low-cost, *in situ* sediment manipulation by the percolation of oxygenated seawater, is capable of increasing the relative abundance, diversity, metabolic capacity and functional potential of microbial communities for aquaculture waste bioremediation. In addition to environmental concerns over organic enrichment and the depletion of dissolved oxygen stemming from the discharge of suspended solid wastes, the potential toxic effects of chemicals used to control and treat disease outbreaks must also be considered^47^. The potential enrichment of functional pathways for xenobiotics degradation and metabolism in the aerobic sediment system demonstrates a potential to bioremediate a variety of chemical therapeutants used in intensive aquaculture, including antibiotics, anaesthetics and anti-parasitic agents. Additional benefits of an aerobic sediment-based treatment system may include the removal of pathogenic bacteria present in the discharge water. Future research should focus on pilot testing deposit-feeder sediment-based effluent treatment systems in conjunction with existing land-based aquaculture to test their ability to treat suspended solid effluent originating from re-circulating aquaculture systems. The next stage would then be to up-scale the treatment systems to determine cost-effective production and expand application of the systems described.

## Methods

### Ethics statement

The study was approved by the Ethics Panels of both Newcastle and Rhodes Universities. No collections were made from wild populations to support this study.

### Rearing conditions and experimental treatments

The study was conducted at HIK Abalone Farm (Pty) Ltd in Hermanus, South Africa (34°26′04.35′′S; 19°13′12.51′′E) between 12^th^ September and 5^th^ December 2012. Study animals were imported from a commercial hatchery in Madagascar in November 2011, quarantined and acclimated as described in Robinson *et al*.^10^. The experimental treatments comprised manipulated sediment systems designed to maintain sediments under either a fully oxic redox regime, hereafter referred to as the ‘oxic’ treatment or under a natural redox-stratified condition, hereafter referred to as the ‘oxic-anoxic’ treatment. The treatments were each allocated to three replicate tanks using a randomised block design.

Six polyethylene tanks with calcium carbonate sediment (125–250 μm particle size) were supplied with aerated, recirculating heated seawater (29.13 ± 0.12 °C). The aeration and tank design used to create the contrasting redox regimes was also as described in Robinson *et al*.^10^. The feed, feeding, and maintenance regimes were as described in Robinson *et al*.^10^; however, the daily feed rations ranged from one to four percent of the total tank biomass per day in the current study. Equitable feed rations were adjusted daily based on sediment quality observations. Epiphytic algae and cyanobacteria were removed monthly, separated, and dried at 50 °C for 48 h. Tanks were subject to a natural photoperiod which increased from 11.45:12.15 L:D to 14.20:09.40 L:D as day length increased over the course of the austral summer.

The sea cucumbers (n = 18) were gut evacuated and weighed prior to stocking into experimental tanks as described previously^10^. Animals with a mean weight of 14.98 ± 0.41 g individual^−1^ (mean ± SE) were randomly allocated to six groups with three individuals per group. Each individual was re-weighed every 28 days over the 84 day experimental period. Growth rate was calculated using wet weight data^10^.

### Water quality, sediment quality and environmental variables

Water quality parameters (temperature, pH, dissolved oxygen, total ammonia nitrogen (NH_4_-N; TAN) and nitrite) were recorded weekly as described in Robinson *et al*.^10^. Additionally, weekly light readings (aerial) were taken using a portable light meter (LX-107, Lutron Electronic Enterprise Co. Ltd, Taipei, Taiwan) positioned 10 cm directly above the tank outflow.

The sediment redox potential was measured on day 84, as described in Robinson *et al*.^10^. Composite samples of the sediment surface layers (upper 2–3 mm) were collected from all tanks to determine sediment parameters. Chlorophyll *a* and phaeopigment concentrations were measured using a variation of the Lorenzen^48^ spectrophotometric method. Extraction was carried out overnight at 4.0 °C with 100% acetone, distilled water was added to return the fluid concentration to 90% acetone before the first spectrophotometric step. Absorbance of 1 ml of the supernatant was read at 665 and 750 nm before and after acidification with 40 μl of 10% HCl against a 90% acetone blank. Sediment samples were dried at 50 °C for 48 h and weighed. The remainder of the composite samples were dried to a constant weight at 50 °C for 48 h and organic carbon and total nitrogen content were analysed prior to, and after, carbonate removal^10^.

### DNA extraction and amplification

Sediment samples were collected from each tank using a one centimetre internal diameter core and sectioned at two centimetre intervals (i.e. from the sediment surface) and at 2.0 and 4.0 cm depths. The coring approach used here does not exclude the possibility of inadvertent transfer of bacteria from one depth to another, for example due to smearing during core insertion and removal. Genomic DNA was extracted from approximately 250 mg of sediment using a DNA isolation kit (PowerSoil™, MoBio, Solana Beach, USA), following manufacturer’s instructions. The variable regions 4 and 5 of the 16 S rRNA gene were amplified using fusion primers, consisting of sequencer specific nucleotides, multiplex identifier tag and template-specific nucleotides with the template-specific sequences within the forward and reverse primers respectively; (primer pairs E517F (5′-CAGCAGCCGCGGTAA-3′) and E969–984 (5′-GTAAGGTTCYTCGCGT-3′))^49^. Polymerase chain reaction (PCR) amplification was carried out as follows: a 25 μl PCR mixture consisting of ~5 ng of the extracted genomic DNA, 1X PCR buffer (containing MgCl_2_), 300 μM dNTPs, 10.0 μM of each primer set and 0.5 μl KAPA HiFi HotStart DNA Polymerase (KAPA Biosystems) was subjected to initial enzyme activation and DNA denaturation at 98 °C for five minutes followed by cycling parameters of 98 °C for 45 seconds, 45 °C for 30 seconds, 72 °C for one minute (for five cycles), 98 °C for 45 seconds, 50 °C for 30 seconds, and 72 °C for one minute (20 cycles). A final extension was done at 72 °C for five minutes. The resultant ~540 nt PCR products were gel purified using an AMPure® XP (Beckman Coulter, Ireland) and the double-stranded DNA concentration was determined using PicoGreen® (Invitrogen, Germany) on a Thermo Scientific NanoDrop™ 3300 Fluorospectrometer (Thermo Fisher Scientific, USA). The generated amplicons were pooled in equal amounts and subjected to emulsion PCR before sequencing.

### Pyrosequencing and sequence analysis

Amplicons were sequenced using the GS Titanium Sequencing chemistry (454 Life Sciences, Roche). Reads were de-multiplexed and pre-processed using the automated Roche GS Run Processor pipeline to remove adapter sequences and low quality reads. After de-multiplexing, flowgram data (SFF-files) for each sample were processed using the QIIME Denoiser^50^ according to the QIIME standard protocol^51^. Individual sequences were parsed into sample-specific libraries and screened for reads less than 200 base pairs. Chimera detection was completed using the Chimera Slayer system^52^ in the QIIME 1.8.0 software package. Only sequences flagged as non-chimeras by *de novo* and reference based methods (Greengenes database, August 2013 release)^53^ were retained. Sequences were clustered at 97% similarity and the most abundant sequence was chosen as representative for each operational taxonomic unit (OTU). Taxonomic assignment of the resulting reads was performed using UCLUST^54^ to align sequences against the Greengenes core reference set (August 2013 release^53^) to genus level where possible.

### Statistical and bioinformatics analysis

#### Sea cucumber growth and environmental metadata

Light and water quality data were averaged across the 84 day period to provide a mean value per tank. The mean wet weight of individual *H. scabra* per tank was averaged and the mean value per tank was used for further statistical analysis. Growth and environmental data were tested for homogeneity of variance and for the normal distribution of the residuals using Levene’s and Shapiro Wilk’s tests respectively. All data that met the test assumptions were analysed using a Student t-test at alpha <0.05. Results are expressed as mean ± standard error. Statistical analyses were performed using Statistica version 12.

#### Microbial community composition: Alpha and Beta diversity measures

Sequences were normalized to the minimum number of reads per sample (1,264) and alpha (within samples) diversity parameters were computed using QIIME 1.8.0^51^. Richness estimators included the total number of OTUs observed in a sample (S_obs_) and Chao1^51^, and diversity indices included Shannon and Simpson diversity. Evenness was calculated using the equitability metric defined in QIIME as: (Shannon entropy)/log_2_ (S_obs_). Mean diversity indices were tested for normality using Shapiro Wilk’s test and homogeneity of variance using Levene’s test. Data that met the test assumptions were compared across experimental treatments using mixed-model analysis of variance (ANOVA) to test the effects of redox regime and sediment depth on alpha diversity metrics. Redox regime was included as a fixed factor and sediment depth as a covariate. Significant differences between treatments were identified by Tukey’s honest significant difference (HSD) post-hoc tests. Results are expressed as mean ± standard error and differences considered significant at alpha <0.05. Statistical analyses were performed using Statistica version 12.

Beta diversity analysis was performed on taxonomic data at the phylum level based on a Bray-Curtis dissimilarity matrix to compare community dissimilarities between treatments and separately on the environmental data using principal component analysis (PCA). Analysis was performed in MATLAB^®^ using the PCA function from the FATHOM toolbox^55^. Both tables were normalised to mean zero and standard deviation of one, prior to PCA.

#### Taxonomic groups with statistical differences

As the Proteobacteria had the highest sequence abundance, representing 41.12 ± 1.07% (n = 15) of the total number of sequences, the Proteobacteria sub-classes of Alpha-, Beta-, Delta-, and Gammaproteobacteria were included in all phylum-level analyses. Kruskal-Wallis tests were performed in RStudio^56^ using the KW.R function to identify phyla that were significantly different between treatments. Data were log transformed and differences were considered significant at alpha = 0.01.

#### Linking community analyses to environmental variables

Permutational multivariate analysis of variance using Bray-Curtis distances for microbial community phylum level data was used to quantitatively evaluate the contribution of environmental metadata to the microbial community structure using the ‘adonis’ function in the ‘vegan’ package^57^ in RStudio. As redox regime was the only significant factor further analyses was done by redox regime only.

#### Microbial biomarker discovery and visualization

Quantitative analysis of taxonomic biomarkers sampled at three different depths were identified by Linear Discriminant Analysis Effect Size (LEfSe)^15^ using Kruskal-Wallis Wilcoxon-rank sum tests. Differentially abundant and biologically relevant features were ranked by effect size after undergoing linear discriminant analysis (LDA) with an effect size threshold of 5.0 (on a log10 scale). To further elucidate the functional diversity and metabolic role of the bacterial communities the 86 biomarkers with an LDA score >5.0 were collapsed to reflect the highest level of taxonomic resolution, reducing the number of biomarkers to 24 and 16 in the oxic and oxic-anoxic sediments, respectively. Biomarkers were classified according to their dissimilatory metabolism, oxygen-related eco-physiology and putative functional role based on available literature.

#### Metagenome prediction

Phylogenetic Investigation of Communities by Reconstruction of Unobserved States (PICRUSt) v0.9.0 was applied^16^ to gain further insight into the putative metabolic functions of bacteria differentially enriched in the oxic and oxic-anoxic treatments. The FastTree Greengenes (gg_13_08) phylogeny^53^ annotated with these organisms’ genomes was used to pick closed-reference OTUs based on 65% of the total sequences from the de-multiplexed and quality-filtered reads. Each genus-level OTU was assigned to the Greengenes clade containing the most genomes from that genus and fewest from other genera. Higher-level clades continued with this same assignment pattern. The gene contents were then reconstructed across the GG tree and assigned Kyoto Encyclopaedia of Genes and Genomes (KEGG) Orthology (KO) copy numbers^58^. Each KO entry represents a manually defined ortholog group that corresponds to a node of the KEGG pathway map or module, and which consists of orthologous genes in all available genomes in the KEGG database^58^. The relative abundance of each KO was then estimated per sample by multiplying each OTU abundance by each predicted functional trait abundance. Inferred relative gene abundances were subsequently binned into the six main pathways and their respective functional categories defined by the BRITE hierarchy files that represent the functional hierarchy of KEGG objects. The accuracy of the metagenome predictions was evaluated using weighted Nearest Sequenced Taxon Index (weighted NSTI) scores^16^.

The relative gene counts were analysed in the graphical software package ‘Statistical Analysis of Taxonomic and Functional Properties’ (STAMP)^59^. To simplify analysis any non-microbial categories, for example ‘Human Diseases’ were excluded from further analysis. Metabolic pathways that were significantly different between treatments were identified using two-sided Welch’s t-tests comparing gene counts at levels 2 and 3 of the BRITE hierarchies with a Bonferroni multiple test correction to control for false discovery rate. Only pathways or modules with a significantly different mean proportion of gene counts between treatments are presented (alpha = 0.05).

## Additional Information

**How to cite this article**: Robinson, G. *et al*. Profiling bacterial communities associated with sediment-based aquaculture bioremediation systems under contrasting redox regimes. *Sci. Rep.* **6**, 38850; doi: 10.1038/srep38850 (2016).

**Publisher's note:** Springer Nature remains neutral with regard to jurisdictional claims in published maps and institutional affiliations.

## Supplementary Material

Supplementary Information

## Figures and Tables

**Table 1:** Mean (±standard error) values for the environmental parameters recorded over the 84 day experimental period in sea cucumber tanks subjected to oxic-anoxic and oxic redox regimes.

Parameter	Oxic-anoxic			Oxic			t-value	p
	Mean		SE	Mean		SE		
Light (Lux)	1 406.67	±	119.21	1 465.67	±	217.13	−0.24	0.8234
Temperature (°C)	29.81	±	0.01	29.13	±	0.12	5.84	0.0043*
pH	8.39	±	0.01	8.33	±	0.02	2.30	0.0831
Dissolved oxygen (mg L^−1^)	7.49	±	0.11	7.89	±	0.06	−3.15	0.0347*
Dissolved oxygen (%)	116.00	±	1.64	121.33	±	1.54	−2.37	0.0770
Ammonia (μg L^−1^)	17.79	±	2.33	19.15	±	5.17	−0.24	0.8225
Nitrite (μg L^−1^)	15.48	±	1.13	15.61	±	1.02	−0.08	0.9383
Chlorophyll *a* (μg g^−1^)	2.05	±	0.75	2.43	±	0.57	−0.41	0.7038
Phaeopigment (μg g^−1^)	0.21	±	0.08	0.39	±	0.09	−0.61	0.5738
Dry weight green macroalgae (g)	12.74	±	3.24	7.19	±	2.81	1.29	0.2655
Dry weight cyanobacteria (g)	99.66	±	2.72	221.61	±	34.95	−3.48	0.0254*
Redox potential (mV)	−188.42	±	11.52	33.50	±	11.00	−13.93	0.0002*
Organic carbon (%)	1.59	±	0.32	1.58	±	0.13	0.03	0.9781
Total nitrogen (%)	0.06	±	0.02	0.08	±	0.01	−0.74	0.4982
C:N (%)	26.03	±	2.32	21.19	±	3.69	1.04	0.3562

A student’s t test was performed to identify significant differences, p = 0.05 (indicated by an asterisk*).

**Table 2:** Mean (±standard error) values for alpha diversity measures computed in QIIME for bacterial communities present at three different depths in the sediment of sea cucumber culture tanks subjected to contrasting oxic-anoxic and oxic redox regimes.

	Oxic-anoxic									Oxic									Redox regime	Depth
	0 cm			2 cm			4 cm			0 cm			2 cm			4 cm				
No. of reads	3751.50	±	35.50	3600.00	±	1501.60	3962.00	±	1352.95	2765.33	±	198.95	2602	±	365.54	2572	±	—	ns	ns
Sobs	254.55	±	32.00^a^	247.77	±	14.86^a^	268.73	±	38.04^a^	357.13	±	11.49^a^	472.4	±	14.76^b^	565.7	±	-^b^	p < 0.001	ns
Chao 1	380.98	±	49.46^a^	393.97	±	39.60^a^	410.91	±	65.97^a^	542.84	±	25.10^a,b^	707.23	±	27.18^b,c^	857.68	±	-^c^	p < 0.001	ns
Simpson	0.96	±	0.01^a^	0.96	±	0.01^a^	0.97	±	0.01^a,b^	0.97	±	0.00^a,b^	0.99	±	0.00^b^	0.99	±	-^a,b^	p < 0.001	ns
Shannon	5.73	±	0.39^a,b^	5.70	±	0.13^a^	6.01	±	0.06^a,b^	6.43	±	0.11^b^	7.59	±	0.10^c^	7.94	±	-^c^	p < 0.001	ns
Evenness	0.71	±	0.03^a^	0.72	±	0.03^a^	0.75	±	0.03^a,b^	0.78	±	0.02^a,b^	0.87	±	0.02^b^	0.88	±	-^a,b^	p < 0.001	ns

Chao 1 indicates the number of rare OTUs, Sobs is the observed number of OTUs, the Shannon diversity index combines species richness and evenness, and the Simpson’s dominance index and evenness. A mixed-model ANOVA was performed to identify significant differences between treatments with redox regime included as a fixed factor and depth as a covariate. Tukey HSD post hoc tests were used to evaluate significant results. Different superscript letters within the same row indicate significant differences, p = 0.05.
