# Supplementary material for: Profiling bacterial communities associated with sediment-based aquaculture bioremediation systems under contrasting redox regimes
Source: Sci Rep. 2016 Dec 12;6:38850. doi: 10.1038/srep38850 (PMC5150640; doi:10.1038/srep38850)
Supplement: Supplementary Information [file srep38850-s1.pdf]

Profiling bacterial communities associated with sediment-based aquaculture bioremediation systems under contrasting redox regimes

Georgina Robinson[1,2*], Gary S. Caldwell[1], Matthew J. Wade[3], Andrew Free[4], Clifford L.W. Jones[2], Selina M. Stead[1]

[1]School of Marine Science and Technology, Newcastle University, Newcastle, NE1 7RU, UK.
[2]Department of Ichthyology and Fisheries Science, Rhodes University, Grahamstown 6140, South Africa.
[3]School of Civil Engineering and Geosciences, Newcastle University, Newcastle, NE1 7RU, UK.
[4]Institute of Quantitative Biology, Biochemistry and Biotechnology, School of Biological Sciences, University of Edinburgh, Edinburgh, EH9 3FF, U.K.

Supplementary Figures 1 and 2, and supplementary Tables 1 to 4.

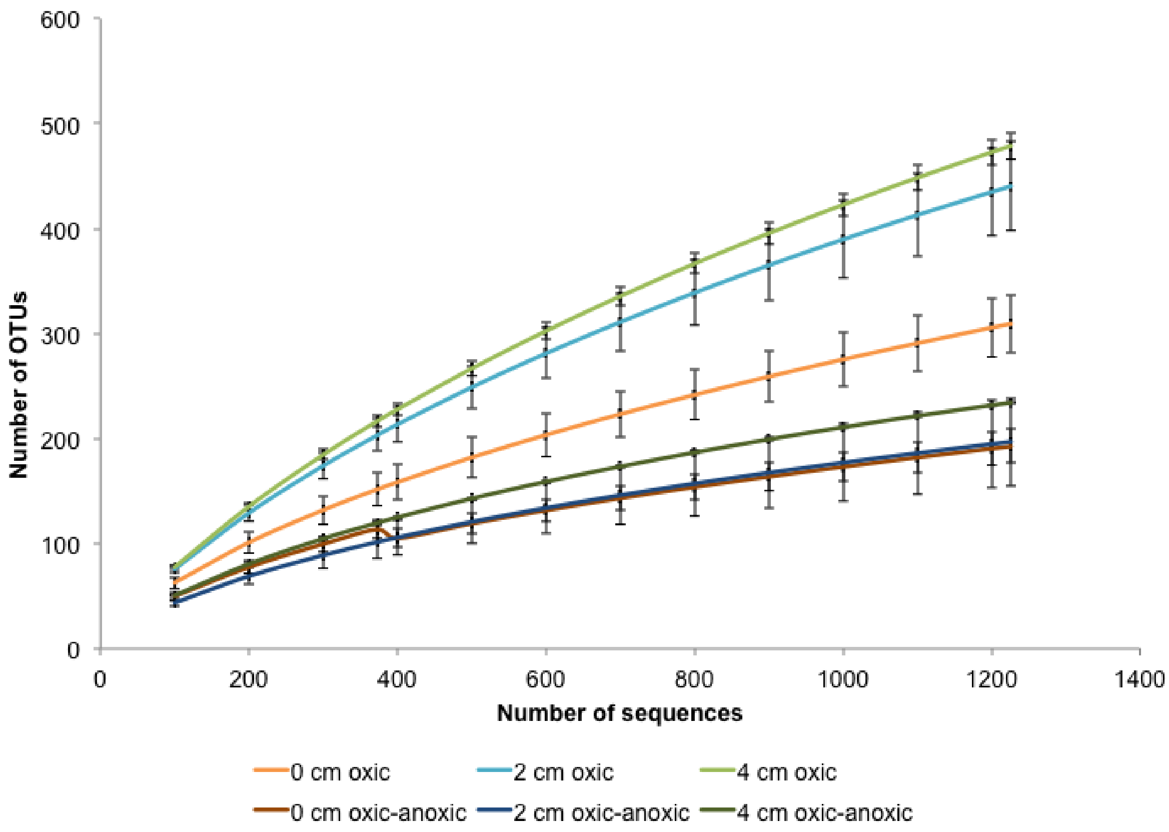

Figure S1. Rarefaction curves of bacterial 16S rRNA gene sequences recovered from the three depths (0, 2 and 4 cm) of sediment taken from *Holothuria scabra* culture tanks subjected to a fully oxic and a stratified oxic-anoxic redox regime. Each line represents the mean of treatment replicates (n = 3).

Table S1. Quantitative contribution of environmental parameters to bacterial community structure determined by permutational multivariate analysis of variance using distance Matrices (permanova).

	df	SS	Mean squares	F model	R^2	p
Redox potential	1	0.33417	0.33417	15.2182	0.58837	0.001
Dissolved oxygen	1	0.00712	0.00712	0.3243	0.01254	0.784
Light	1	0.02019	0.02019	0.9193	0.03554	0.411
Temperature	1	0.00205	0.00205	0.0933	0.00361	0.971
pH	1	0.0068	0.0068	0.3099	0.01198	0.819
Residuals	9	0.19763	0.02196		0.34796	
Total	14	0.56796			1	

Table S2. Classification of the taxonomic biomarkers identified by Linear Discriminant Effect Size Analysis (LEfSE) in oxic sediments according to their oxygen-related eco-physiology and dissimilatory metabolism.

Metabolism & redox zone			Phylum	Class	Order	Family	Genus	Relationship to O_2	Ecological role	Ref
Heterotophic	Aerobic respiration	Oxic	Bacteroidetes	Cytophagia	Cytophagales	Flammeovirgaceae		Aerobe	Oxidation: carbohydrates	[1]
			Bacteroidetes	[Rhodothermi]	[Rhodothermales]	Rhodothermaceae		Aerobe	Oxidation: sugars & amino acids	[2]
			Planctomycetes	OM190	CL500_15			Aerobe		
			Planctomycetes	Phycisphaerae	Phycisphaerales			Aerobe	Oxidation: sugars & sugar alcohols	[3]
			Planctomycetes	Planctomycetia	Pirellulales	Pirellulaceae		Aerobe		
			Planctomycetes	Planctomycetia	Planctomycetales	Planctomycetaceae	Planctomyces	Aerobe or facultative anaerobe	Oxidation: carbohydrates	[4]
			Proteobacteria	δ-proteobacteriaγ	Myxococcales	Nannocystaceae	Plesiocystis	Obligate aerobe	Proteolytic/bacteriolytic	[5]
			Proteobacteria	γ-proteobacteria	Alteromonadales	Alteromonadaceae	Alteromonas	Aerobe	Oxidation: carbohydrates, alcohols, organic & amino acids.	[6]
			Proteobacteria	γ-proteobacteria	Alteromonadales	Alteromonadaceae	nsmpVI18	Aerobe	Degradation: ι-carrageenan, methylthiopropanoate	[7]
			Proteobacteria	γ-proteobacteria	Legionellales	Coxiellaceae		Aerobe	Obligate intracellular parasite	[8]
			Verrucomicrobia	Verrucomicrobiae	Verrucomicrobiales	Verrucomicrobiaceae		Aerobe	Oxidation: mono- & disaccharides	[9]
	Fermentation	Anoxic	Chloroflexi	Anaerolineae	DRC31			Strict anerobe	Chemoorganotrophic, non-photsynethetic	[10, 11]
			Proteobacteria	γ-proteobacteria	Vibrionales	Vibrionaceae	Photobacterium	Facultative anaerobe	D-glucose & D-mannose catabolism with the production of acid	[12]
Methylotrophs			Proteobacteria	γ-proteobacteria	Thiotrichales	Piscirickettsiaceae		Aerobe		[13]
Chemolithotrophic		Oxic	Nitrospirae	Nitrospira	Nitrospirales	Nitrospiraceae		Obligate aerobe	Nitrite oxidation	[14]
		Anoxic	Actinobacteria	Acidimicrobiia	Acidimicrobiales	C111			Ferrous iron (Fe^{2+}) oxidation	[15]
			Proteobacteria	γ-proteobacteria	Thiotrichales	Thiotrichaceae	Thiopilula		Sulphur oxidation	[16]
Phototrophic		Oxic	Cyanobacteria	Chloroplast	Stramenopiles			Aerobe	Oxygenic photosynthesis	
		Anoxic	Proteobacteria	α-proteobacteria	Rhodobacterales	Rhodobacteraceae		Anaerobe	Anoxygenic photosynthesis; oxidation of HS^- to S	[17]
			Proteobacteria	γ-proteobacteria	Chromatiales	Ectothiorhodospiraceae		Anaerobe	Oxidation of HS^- to S	[18]

Table S3. Classification of the taxonomic biomarkers identified by Linear Discriminant Effect Size Analysis (LEfSE) in oxic-anoxic sediments according to their oxygen-related eco-physiology and dissimilatory metabolism.

Metabolism & redox zone			Phylum	Class	Order	Family	Genus	Relationship to O_2	Ecological role	Ref
Heteroptophic	Oxic	Aerobic respiration	Proteobacteria	ε-proteobacteria	Campylobacterales	Helicobacteraceae		Microaerophilic or anaerobic	Reduction: fumarate to succinate	[19]
			Spirochaetes	[Leptospirae]	[Leptospirales]	Sediment-4	SJA-88	Obligate aerobe/ microaerophile	Oxidise long-chain fatty acids or long-chain fatty alcohols	[20]
	Anoxic	Anaerobic respiration	Proteobacteria	δ-proteobacteria	Desulfobacterales	Desulfobacteraceae	Desulfobacter	Strict anaerobe	Reduction: sulphate, sulphite & thiosulphate to H_2S	[21]
			Proteobacteria	δ-proteobacteria	Desulfobacterales	Desulfobulbaceae		Strict anaerobe	Reduction: sulphate to sulphide	[22]
			Tenericutes	Mollicutes				Facultative/ obligate anaerobe	Commensals or parasites	[23]
		Fermentation	Bacteroidetes	Bacteroidia	Bacteroidales	Marinilabiaceae		Facultative anaerobe	Carbohydrate fermentation	[24]
			Bacteroidetes	Bacteroidia	Bacteroidales	SB-1		Anaerobe	Carbohydrate fermentation	[25]
			Chloroflexi	Anaerolineae	Anaerolineales	Anaerolinaceae		Strict anaerobe		[9, 10]
			Firmicutes	Clostridia	Clostridiales	JTB215		Anaerobic to aerotolerant	Cellulolytic	
			Fusobacteria	Fusobacteriia	Fusobacteriales	Fusobacteriaceae	Propionigenium	Strict anaerobe	Decarboxylation of succinate to propionate	[26]
			KSB3	MAT-CR-H3-D11				Strict anaerobe		[27]
			Planctomycetes	Phycisphaerae	AKAU3564			Facultative anaerobe	Fermentation of 5-C and 6-C sugars	[3]
			Spirochaetes	Spirochaetes	Spirochaetales	Spirochaetaceae	Spirochaeta	Obligate or facultative anaerobe	Carbohydrate fermentation	[28]
		Phototrophy	Chlorobi	OPB56				Obligate anaerobe	Anoxygenic photosynthesis; photoheterotoph	[29]

1. Nedashkovskaya, O.I. and W. Ludwig, *Family III. Flammeovirgaceae fam. nov.*, in *Bergey's Manual of Systematic Bacteriology. Volume 4. The Bacteroidetes, Spirochaetes, Tenericutes, (Mollicutes), Acidobacteria, Fibrobacteres, Fusobacteria, Dictyoglomi, Gemmatimonadetes, Lentisphaerae, Verrucomicrobia, Chlamydiae and Planctomycetes*, N.R. Krieg, et al., Editors. 2010, Springer: New York. p. 442.

2. Ludwig, W., J. Euzéby, and W.B. Whitman, *Family I. Rhodothermaceae fam. nov.*, in *Bergey's Manual of Systematic Bacteriology. Second Edition. Volume 4. The Bacteroidetes, Spirochaetes, Tenericutes, (Mollicutes), Acidobacteria, Fibrobacteres, Fusobacteria, Dictyoglomi, Gemmatimonadetes, Lentisphaerae, Verrucomicrobia, Chlamydiae and Planctomycetes*, N.R. Krieg, et al., Editors. 2010, Springer: New York. p. 457.
3. Youssef, N. and M. Elshahed, *The Phylum Planctomycetes*, in *The Prokaryotes*, E. Rosenberg, et al., Editors. 2014, Springer Berlin Heidelberg. p. 759-810.
4. Ward, N.L., J.T. Staley, and J.M. Schmidt, *Genus I. Planctomyces* in *Bergey's Manual of Systematic Bacteriology, second edition, vol. 4 The Bacteroidetes, Spirochaetes, Tenericutes, (Mollicutes), Acidobacteria, Fibrobacteres, Fusobacteria, Dictyoglomi, Gemmatimonadetes, Lentisphaerae, Verrucomicrobia, Chlamydiae, and Planctomycetes*, N.R. Krieg, et al., Editors. 2010, Springer: New York. p. 881-895.
5. Reichenbach, H., *Family IV. Nannocystaceae fam. nov.*, in *Bergey's Manual of Systematic Bacteriology, second edition, vol. 2 (The Proteobacteria), part C (The Alpha-, Beta-, Delta-, and Epsilonproteobacteria)*, D.J. Brenner, et al., Editors. 2005, Springer: New York. p. Accessed online.
6. Bowman, J.P. and T.A. McMeekin, *Genus I. Alteromonas Baumann, Baumann, Mandel and Allen 1972, 418, emend. authier, Gauthier and Christen 1995a, 760*, in *Bergey's Manual of Systematic Bacteriology, second edition, vol. 2 (The Proteobacteria), part B (The Gammaproteobacteria)*, D.J. Brenner, N.R. Krieg, and J.T. Staley, Editors. 2005, Springer: New York.
7. Bowman, J.P. and T.A. McMeekin, *Order X. Alteromonadales ord. nov.*, in *Bergey's Manual of Systematic Bacteriology, second edition, vol. 2 (The Proteobacteria), part B (The Gammaproteobacteria)*, D.J. Brenner, N.R. Krieg, and J.T. Staley, Editors. 2005, Springer: New York. p. 443.
8. Garrity, G.M., J.A. Bell, and L. T., *Family II. Coxiellaceae fam. nov.*, in *Bergey's Manual of Systematic Bacteriology, second edition, vol. 2 (The Proteobacteria), part B (The Gammaproteobacteria)*, D.J. Brenner, N.R. Krieg, and J.T. Staley, Editors. 2005, Springer: New York. p. 237.
9. Hedlund, B.P., *Family I. Verrucomicrobiaceae* in *Bergey's Manual of Systematic Bacteriology, second edition, vol. 4 The Bacteroidetes, Spirochaetes, Tenericutes, (Mollicutes), Acidobacteria, Fibrobacteres, Fusobacteria, Dictyoglomi, Gemmatimonadetes, Lentisphaerae, Verrucomicrobia, Chlamydiae, and Planctomycetes*, N.R. Krieg, et al., Editors. 2010. p. 803.
10. Yamada, T., et al., *Anaerolinea thermolimosa sp. nov., Levilinea saccharolytica gen. nov., sp. nov. and Leptolinea tardivitalis gen. nov., sp. nov., novel filamentous anaerobes, and description of the new classes Anaerolineae classis nov. and Caldilineae classis nov. in the bacterial phylum Chloroflexi.* International Journal of Systematic and Evolutionary Microbiology, 2006. **56**(6): p. 1331-1340.
11. Wrighton, K.C., et al., *Metabolic interdependencies between phylogenetically novel fermenters and respiratory organisms in an unconfined aquifer.* ISME J, 2014. **8**(7): p. 1452-1463.

12. Thyssen, A. and F. Ollevier, *Genus II. Photobacterium* in *Bergey's Manual of Systematic Bacteriology, second edition, vol. 2 (The Proteobacteria), part B (The Gammaproteobacteria)*, D.J. Brenner, N.R. Krieg, and J.T. Staley, Editors. 2005, Springer: New York. p. 546-552.
13. Fryer, J.L. and C.N. Lannan, *Family II. Piscirickettsiaceae fam. nov.*, in *Bergey's Manual of Systematic Bacteriology, second edition, vol. 2 (The Proteobacteria), part B (The Gammaproteobacteria)*, D.J. Brenner, N.R. Krieg, and J.T. Staley, Editors. 2005, Springer: New York. p. 180.
14. Daims, H., *The Family Nitrospiraceae*, in *The Prokaryotes*, E. Rosenberg, et al., Editors. 2014, Springer Berlin Heidelberg. p. 733-749.
15. Norris, P.R., *Order I. Acidimicrobiales* in *Bergey's Manual of Systematic Bacteriology. Vol. 5, the Actinobacteria.*, D. Bergey, et al., Editors. 2012, Springer: New York. p. 1969.
16. Jones, D.S., B.E. Flood, and J.V. Bailey, *Metatranscriptomic analysis of diminutive Thiomargarita-like bacteria ("Candidatus Thiopilula" spp.) from abyssal cold seeps of the barbados accretionary prism.* Applied and Environmental Microbiology, 2015. **81**(9): p. 3142-3156.
17. Pujalte, M.J., et al., *The Family Rhodobacteraceae*, in *The Prokaryotes - Alphaproteobacteria and Betaproteobacteria*, E. Rosenberg, et al., Editors. 2014, Springer Verlag. p. 439-512.
18. Imhoff, J.F., *Family II. Ectothiorhodospiraceae* in *Bergey's Manual of Systematic Bacteriology, second edition, vol. 2 (The Proteobacteria), part B (The Gammaproteobacteria)*, D.J. Brenner, N.R. Krieg, and J.T. Staley, Editors. 2005, Springerq: New York. p. 41-43.
19. Garrity, G.M., J.A. Bell, and T. Lilburn, *Family II. Helicobacteraceae fam. nov.*, in *Bergey's manual of systematic bacteriology. Vol. 2. The proteobacteria. Part C. The alpha-, beta-, delta-, and epsilonproteobacteria*, D.J. Brenner and J.T. Staley, Editors. 2005, Springer: New York.
20. Gupta, R.S., S. Mahmood, and M. Adeolu, *Erratum: A phylogenomic and molecular signature based approach for characterization of the Phylum Spirochaetes and its major clades: proposal for a taxonomic revision of the phylum.* Frontiers in Microbiology, 2013. **4**: p. 322.
21. Kuever, J., F.A. Rainey, and F. Widdel, *Genus I. Desulfobacter* in *Bergey's manual of systematic bacteriology. Vol. 2. The proteobacteria. Part C. The alpha-, beta-, delta-, and epsilonproteobacteria*, D.J. Brenner and J.T. Staley, Editors. 2005, Springer: New York.
22. Kuever, J., F.A. Rainey, and F. Widdel, *Family II. Desulfobulbaceae fam. nov.*, in *Bergey's manual of systematic bacteriology. Vol. 2. The proteobacteria. Part C. The alpha-, beta-, delta-, and epsilonproteobacteria*, D.J. Brenner and J.T. Staley, Editors. 2005, Springer: New York.
23. Brown, D.R., et al., *Class I. Mollicutes*, in *Bergey's Manual of Systematic Bacteriology, second edition, vol. 4 The Bacteroidetes, Spirochaetes, Tenericutes, (Mollicutes), Acidobacteria, Fibrobacteres, Fusobacteria, Dictyoglomi, Gemmatimonadetes, Lentisphaerae, Verrucomicrobia, Chlamydiae, and Planctomycetes*, N.R. Krieg, et al., Editors. 2010, Springer: New York. p. 568-573.
24. Ludwig, W., J. Euzéby, and W. Whitman, *Family II. Marinilabiliaceae fam. nov.*, in *Bergey's Manual of Systematic Bacteriology, second edition, vol. 4 The Bacteroidetes, Spirochaetes, Tenericutes, (Mollicutes), Acidobacteria, Fibrobacteres, Fusobacteria, Dictyoglomi,*

Gemmatimonadetes, Lentisphaerae, Verrucomicrobia, Chlamydiae, and Planctomycetes, N.R. Krieg, et al., Editors. 2010, Springer: New York. p. 49.
25. Krieg, N.R., *Order I. Bacteroidales ord. nov.*, in *Bergey's Manual of Systematic Bacteriology, second edition, vol. 4 The Bacteroidetes, Spirochaetes, Tenericutes, (Mollicutes), Acidobacteria, Fibrobacteres, Fusobacteria, Dictyoglomi, Gemmatimonadetes, Lentisphaerae, Verrucomicrobia, Chlamydiae, and Planctomycetes*, N.R. Krieg, et al., Editors. 2010, Springer: New York. p. 25.
26. Schink, B. and P.H. Janssen, *Genus IV. Propionigenium* in *Bergey's Manual of Systematic Bacteriology, second edition, vol. 4 The Bacteroidetes, Spirochaetes, Tenericutes, (Mollicutes), Acidobacteria, Fibrobacteres, Fusobacteria, Dictyoglomi, Gemmatimonadetes, Lentisphaerae, Verrucomicrobia, Chlamydiae, and Planctomycetes*. 2010, Springer: New York. p. 761.
27. Sekiguchi, Y., et al., *First genomic insights into members of a candidate bacterial phylum responsible for wastewater bulking.* PeerJ, 2015. **3**: p. e740.
28. Leschine, S. and B.J. Paster, *Genus I. Spirochaeta* in *Bergey's Manual of Systematic Bacteriology, second edition, vol. 4 The Bacteroidetes, Spirochaetes, Tenericutes, (Mollicutes), Acidobacteria, Fibrobacteres, Fusobacteria, Dictyoglomi, Gemmatimonadetes, Lentisphaerae, Verrucomicrobia, Chlamydiae, and Planctomycetes*, N.R. Krieg, et al., Editors. 2010, Springer: New York. p. 473-483.
29. Hiras, J., et al., *Refining the phylum Chlorobi by resolving the phylogeny and metabolic potential of the representative of a deeply branching, uncultivated lineage.* ISME Journal, 2015.

Table S4. Accuracy of the predicted metagenomes for samples evaluated by Nearest Sequenced Taxon Identify (NSTI) score and sequence identity (calculated as 1-NSTI score). The NSTI scores refer to the average branch length that separates OTUs in the sample from the reference genome, weighted by the abundance of the OTU in the sample; thus low NSTI scores refer to shorter branch lengths and indicate a more accurate prediction. Data are presented as means ± SE (n = 3 except for treatments 'oxic-anoxic 0 cm' and 'oxic 4 cm' where replicates were lost).

**Redox regime**	**Sediment depth**	**NSTI score**			**Sequence identity (%)**		
Oxic-anoxic	0 cm	0.14	±	0.01	0.86	±	0.01
Oxic-anoxic	2 cm	0.14	±	0.00	0.86	±	0.00
Oxic-anoxic	4 cm	0.13	±	0.01	0.87	±	0.01
Oxic	0 cm	0.20	±	0.01	0.80	±	0.01
Oxic	2 cm	0.18	±	0.00	0.82	±	0.00
Oxic	4 cm	0.18			0.82		

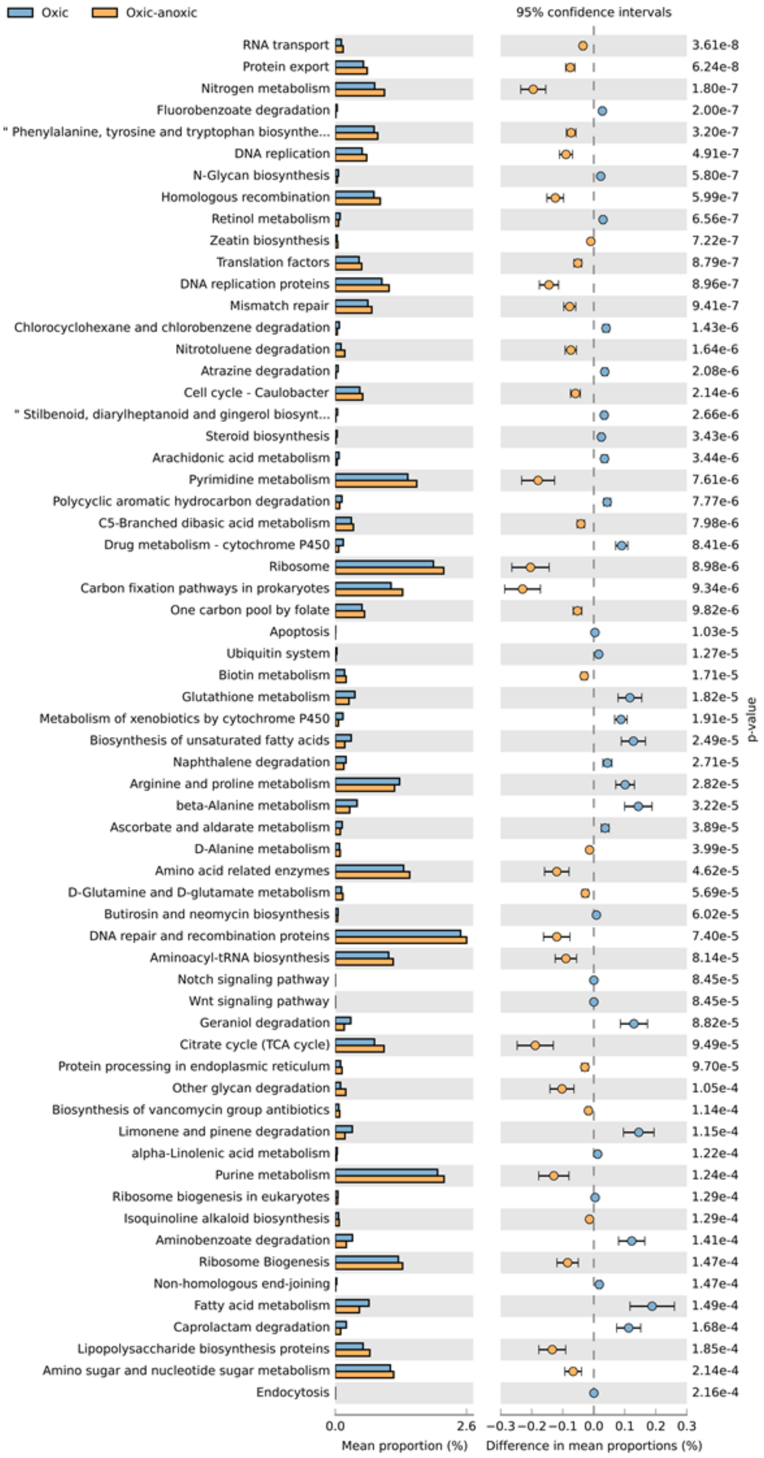

Figure S2. Extended error bar plot showing the mean proportion (%) and the difference in the mean proportion of gene counts at level three of the BRITE functional hierarchy between oxic-anoxic and oxic treatments with 95 % confidence intervals. Significant differences in gene abundances were determined using two-sided Welch's t-tests (alpha = 0.05) with a Bonferroni multiple test correction to control for false discovery rate.
